# Health inequities in the diagnosis and outcome of sepsis in Argentina: a prospective cohort study

**DOI:** 10.1186/s13054-019-2522-6

**Published:** 2019-07-09

**Authors:** Elisa Estenssoro, Cecilia I. Loudet, Vanina S. K. Edul, Javier Osatnik, Fernando G. Ríos, Daniela N. Vásquez, Mario O. Pozo, Bernardo Lattanzio, Fernando Pálizas, Francisco Klein, Damián Piezny, Paolo N. Rubatto Birri, Graciela Tuhay, Anatilde Díaz, Analía Santamaría, Graciela Zakalik, Arnaldo Dubin, investigators of the SATISEPSIS Group, Carolina Enrico, Mariel Romitelli, Mariel A. García, José Celia, Leandro Machuca, Fernando Pálizas, Mario Pozo, Bernardo Latanzio, Emanuel Valgolio, Mario Kenar, Carlos Sosa, Sergio Sarquis, Graciela Tuhay, Francisco Klein, Ariel Sosa, Daniel Ivulich, Luciana Bianchi, Enrique Correger, Carla Groer, Victoria Arrosagaray, Graciela Cueto, Carlos Cozzani, Gustavo Badariotti, Bernardo de Diego, Daniela N. Vazquez, Gustavo Plotnikov, Analía Santa-María, Mariana Bertes, Alejandro Gomez, María S. Santagiuliana, Margarita Tavela, Pierina Bachetti, Célica Irrazabal, Alejandro Risso-Vazquez, Paolo N. Rubatto-Birri, Gabriel Olarte, Veronica M. Cannatelli, Anatilde Díaz, Analía García, Estefanía Minoldo, Cayetano Galletti, Esteban Payer, Marcelo Avilez, Silvio E. Lazzeri, Luis A. Huespe, Lorena A. Parra, Fernando Kurban, Carlos A. Pellegrini, Adrian A. Martin, Graciela Zakalik, Magalí Sanchez, Natalia Barreto, Alfredo E. Carreras, Joahana Bastias, Julián Ivacachi, María L. Campassi, Fabio G. Repetto, María G. Saenz, Cecilia Marchena, María R. Marino, Gerardo Ezcurra, Sebastián Caravaggio, María A. García, Ana M. Mazzola, Analía Piernatei, Estela Molinas, Mauro Iadanza, Mario A. Traba, Leda F. Bacci, Adriana Fernandez, Damián Piezny, Constanza Arias, Gustavo Chaparro, Graciela C. Lopez, Agustín Fernández, Catalina Reyes-Najera, Adriana Baldiviezo, Alejandra Flores, Alejandro Risso-Vazquez, Irma Moyano, Mónica Quinteros, Laura Budrovich, Lilen Corzo, Sebastián A. Amieva, Melisa Ré, Nicolás Rocchetti, Juan C. Pendino, Lisandro Bettini, Lionel Talamonti, Gustavo Izaguirre, German Schmukler, Ignacio Sabbag, Tomas F. Diez, Laura Bergallo, Cecilia González, Carlos Lovezio, Daniel Duarte, Romina Nicastro, Fernando Bertoletti, Esteban Milioto

**Affiliations:** 1 Servicio de Terapia Intensiva, Hospital Interzonal de Agudos San Martin de La Plata, Calle 42 No.577, 1900 La Plata, Buenos Aires Argentina; 2 Hospital Juan A Fernández, Buenos Aires, Argentina; 3 Hospital Alemán, Buenos Aires, Argentina; 4 Hospital Alejandro Posadas, El Palomar, Buenos Aires, Argentina; 5 Sanatorio Anchorena, Buenos Aires, Argentina; 6 Clínica Bazterrica, Buenos Aires, Argentina; 7 Clínica Santa Isabel, Buenos Aires, Argentina; 8 Hospital Universitario Fundación Favaloro, Buenos Aires, Argentina; 9 Sanatorio Otamendi y Miroli, Buenos Aires, Argentina; 10 Hospital Misericordia, Córdoba, Argentina; 11 Sanatorio de la Trinidad Mitre, Buenos Aires, Argentina; 12 Hospital Lagomaggiore, Mendoza, Argentina

**Keywords:** Sepsis-3, Septic shock, Health inequity, Sepsis awareness, Socioeconomic status, SES

## Abstract

**Background:**

Socioeconomic variables impact health outcomes but have rarely been evaluated in critical illness. Low- and middle-income countries bear the highest burden of sepsis and also have significant health inequities. In Argentina, public hospitals serve the poorest segment of the population, while private institutions serve patients with health coverage. Our objective was to analyze differences in mortality between public and private hospitals, using Sepsis-3 definitions.

**Methods:**

This is a multicenter, prospective cohort study including patients with sepsis admitted to 49 Argentine ICUs lasting 3 months, beginning on July 1, 2016. Epidemiological, clinical, and socioeconomic status variables and hospital characteristics were compared between patients admitted to both types of institutions.

**Results:**

Of the 809 patients included, 367 (45%) and 442 (55%) were admitted to public and private hospitals, respectively. Those in public institutions were younger (56 ± 18 vs. 64 ± 18; *p* < 0.01), with more comorbidities (Charlson score 2 [0–4] vs. 1 [0–3]; *p* < 0.01), fewer education years (7 [7–12] vs. 12 [10–16]; *p* < 0.01), more frequently unemployed/informally employed (30% vs. 7%; *p* < 0.01), had similar previous self-rated health status (70 [50–90] vs. 70 [50–90] points; *p* = 0.30), longer pre-admission symptoms (48 [24–96] vs. 24 [12–48] h; *p* < 0.01), had been previously evaluated more frequently in any healthcare venue (28 vs. 20%; *p* < 0.01), and had higher APACHE II, SOFA, lactate levels, and mechanical ventilation utilization. ICU admission as septic shock was more frequent in patients admitted to public hospitals (47 vs. 35%; *p* < 0.01), as were infections caused by multiresistant microorganisms. Sepsis management in the ICU showed no differences. Twenty-eight-day mortality was higher in public hospitals (42% vs. 24%; *p* < 0.01) as was hospital mortality (47% vs. 30%; *p* < 0.01). Admission to a public hospital was an independent predictor of mortality together with comorbidities, lactate, SOFA, and mechanical ventilation; in an alternative prediction model, it acted as a correlate of pre-hospital symptom duration and infections caused by multiresistant microorganisms.

**Conclusions:**

Patients in public hospitals belonged to a socially disadvantaged group and were sicker at admission, had septic shock more frequently, and had higher mortality. Unawareness of disease severity and delays in the health system might be associated with late admission. This marked difference in outcome between patients served by public and private institutions constitutes a state of health inequity.

**Electronic supplementary material:**

The online version of this article (10.1186/s13054-019-2522-6) contains supplementary material, which is available to authorized users.

## Introduction

While there is a significant body of knowledge about socioeconomic determinants of health in chronic disease and in all-cause mortality [1–4], how these factors impact the outcomes of critical illness still remains mostly unknown. The information, however, is slowly beginning to emerge: it has been demonstrated that decreasing Gross National Income (GNI) per capita is associated with rising mortality in ICU patients in general and also in patients with ARDS [5–7]. Sepsis is responsible for approximately 5.3 million deaths annually worldwide, but this data likely underestimates true global figures as it is extrapolated from high-income countries (HICs). Low- and middle-income countries (LMICs), the most populated regions of the world, bear the highest burden of sepsis [8–10]. LMICs face their own challenges: microorganisms causing sepsis differ from those of high-income countries, clinical outcomes might be poorer, and in LMICs, the provision of critical care could be suboptimal [10–15]. Most importantly, within these countries, profound inequities, defined as systematic, unjust, and preventable differences in determinants of health, such as socioeconomic status (SES), demographics, and geography, might create a health gradient that ultimately affects outcomes for various population subgroups [16].

Additionally, health systems are usually fragmented, consisting of an overloaded public sector serving the poor, social security agencies for formal workers, and a private sector for the wealthy [17–20]. This complex structure leads to further social segregation, given that the healthcare process perpetuates the differences in SES.

The SATISEPSIS prospective cohort study in 49 ICUs in Argentina validated Sepsis-3 definitions [21] in an upper middle-income country and showed epidemiologic characteristics, management, outcomes, and predictors of mortality [22]. A key secondary purpose of SATISEPSIS was to examine differences in outcomes and their determinants in patients admitted to the different health subsectors, which is explored in this study. Our hypothesis was that higher mortality, usually ascribed to admission to public hospitals [23–25], was attributable to a more advanced—and, as such, more severe—state of the disease on admission. We also sought to identify the independent prognostic factors that could lead to this negative outcome.

Some of the results of these studies have been previously reported in the form of an abstract [26].

## Methods

Detailed methods and the main results for SATISEPSIS have been published elsewhere [22]. Briefly, SATISEPSIS was a national, multicenter, prospective cohort study lasting 3 months, beginning on July 2, 2016. The study was conceived by the Argentine Society of Intensive Care (SATI: Sociedad Argentina de Terapia Intensiva) and partially sponsored by the Argentine National Ministry of Health. The primary aims were to characterize the epidemiology and outcome of sepsis in an upper middle-income country using the Sepsis-3 definitions and to assess its prognostic validity. The study was approved by each hospital’s Institutional Review Board, and informed consent was signed by patients or their relatives.

Patients included were ≥ 18 years, admitted to participating ICUs with a suspected infection that triggered blood cultures and/or other body fluid sampling, and administration of antibiotics within 24 h. Patients with infections developed during ICU stay were also considered.

Patients were assigned to the categories of infection, sepsis, or septic shock according to Sepsis-3 definitions [21].

On ICU admission, recorded data were epidemiological data, Charlson score, self-rated overall health status previous to the current disease (Euro-QoL visual analogue scale [EQ-VAS], from 100 points [best possible status] to 0) [27], duration of infection symptoms, home-to-hospital distance, years of education, occupation, previous evaluation in another healthcare venue for the current disease, APACHE II and SOFA scores [28], presence of systemic inflammatory response syndrome (SIRS), presumed site of infection, and origin of infection. If patients were unable to respond, data were provided by the next of kin. Other measured variables were serum lactate; vasopressor use; fluid requirement; time to administration of antimicrobials and adequacy, according to isolated microorganisms; presence of highly resistant microorganisms; and organizational characteristics of each participating center.

For this study, the main comparisons were between patients admitted to public or private hospitals. Public institutions comprise two sector blocks: hospitals dependent on the national or provincial Ministry of Health and others belonging to the social security system; they serve the poorest segment of the population and the formal workers, respectively [29]. For this analysis, they were merged into the “Public” category, as has been suggested [19]. In contrast, private hospitals are for-profit or non-profit privately funded institutions, which serve patients with private health insurance or who pay for services out of pocket. The primary outcome was hospital mortality.

### Statistical analysis

Data are presented as proportions, mean and standard deviations, or median and [0.25–0.75] percentiles. Differences between patients admitted to public and private hospitals were analyzed with chi-square or Fisher’s tests or *t* or Wilcoxon rank sum tests, as appropriate. Our aim was to determine if there was an association between mortality and the type of hospital. Variables differing between survivors and nonsurvivors with a *p* < 0.10 in bivariable analysis were entered into two different multivariable logistic regression models, one which included the public/private category and the other which did not. Calibration was assessed using the Hosmer-Lemeshow test. A receiver operating characteristic (ROC) curve was constructed to evaluate model discrimination. To avoid bias introduced by missing data and assuming they were missing at random, the analysis of the primary outcome was replicated after multiple imputation.

Data were analyzed with Stata 14.0 (StataCorp LP, College Station, TX, USA).

## Results

**Fig. 1:**
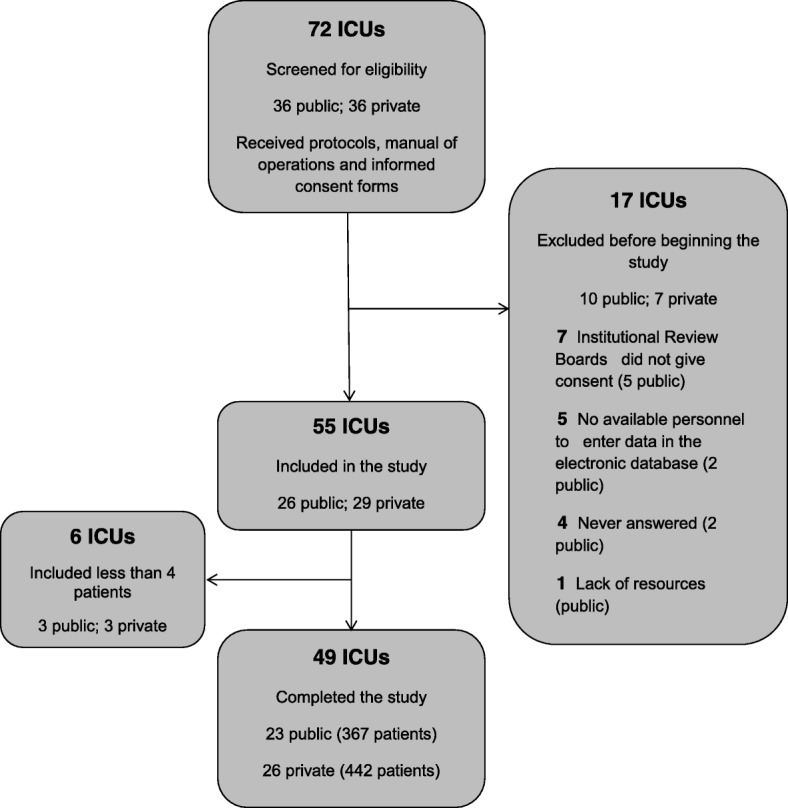
Flow chart of the SATISEPSIS study and distribution of ICUs according to their location in public and private hospitals

Overall recruitment was 809 patients. Of the 49 participating ICUs, 23 (43%) belonged to the public sector and 26 (57%) to the private, and included 367 (45%) and 442 (55%) patients, respectively (Fig. 1). Only 2 of the 23 hospitals of the public system corresponded to the social security system.

Compared to patients in private hospitals, patients in public institutions were significantly younger (56 ± 18 vs. 64 ± 18; *p* < 0.01), had lower BMI (26 [23–29] vs. 27 [24–31]; *p* < 0.01), had more comorbidities (Charlson score 2 [0–4] vs. 1 [0–3] points; *p* < 0.01), had alcohol-related problems (22% vs. 7%; *p* < 0.01), had fewer years of education (7 [7–12] vs. 12 [10–16]; *p* < 0.01), and were more frequently unemployed or had illegal jobs (30% vs. 7%; *p* < 0.01). The perception of the self-rated overall health status prior to the present disease was similar, according to EQ-VAS. With respect to sepsis/septic shock, the duration of pre-admission symptoms was twice that of patients in public hospitals (48 [24–96] vs. 24 [12–48] h; *p* < 0.01), and they had been evaluated more frequently in any healthcare venue before admission (28% vs. 20%; *p* < 0.01), compared to patients in private hospitals. Home-to-hospital distances were similar for both groups. All comparisons are shown in Table 1.

**Table 1:** Patient epidemiological characteristics, socioeconomic status variables, perception of previous health status, and duration of symptoms before admission

	Public hospitals *N* = 23	Private hospitals *N* = 26	Missing values	*p* value
Number of patients	367 (45)	442 (55)		
Age (years)	56 ± 18 *N* = 367	64 ± 18 *N* = 442	0	< 0.01
Female gender	157/367 (43)	197/442 (55)	0	0.61
Body mass index (kg/m^2^)	26 [23–29] *N* = 341	27 [24–31] *N* = 426	42	< 0.01
Years of education	7 [7–12] *N* = 226	12 [10–16] *N* = 326	257	< 0.01
Occupation^§^			0	< 0.01
Legally employed or health-insured	241/367 (66)	397/442 (90)		
Illegal worker or unemployed	126/367 (34)	45/442 (10)		
Charlson score	2 [0–4] *N* = 361	1 [0–3] *N* = 433	15	< 0.01
Self-perception of previous health state EQ-VAS*	70 [50–90] *N* = 226	70 [50–90] *N* = 350	233	0.30
Smoking habit	110/358 (31)	114/430 (27)	21	0.19
Alcohol-related problem	77/358 (22)	30/430 (7)	21	< 0.01
Distance to the hospital (km)	0 [0–10] *N* = 353	1 [0–10] *N* = 431	25	0.13
Duration of sepsis symptoms (hours)	48 [24–96] *N* = 264	24 [12–48] *N* = 360	185	< 0.01
Previous evaluation in any healthcare venue^ǁ^	98/356 (28)	85/433 (20)	20	< 0.01

Data are presented as *n* (%), mean ± standard deviation, or mdn [0.25–0.75] percentiles, unless specified

^§^The occupation category of “legally employed or health-insured” comprises patients with legal jobs, students, retired, and homemakers

*EQ-VAS: EuroQol visual analogue scale (from 100 points [best health state to 0 worst] self-evaluated health state, previously to the diagnosis of sepsis)

^ǁ^Includes primary practices, lower-complexity hospitals, or major hospitals

Patients admitted to public hospitals had higher APACHE II, septic shock, requirement of mechanical ventilation, and lactate levels than patients in private hospitals (*p* < 0.01 in all cases) (Table 2). There were no differences between groups in time to receiving the first antibiotic dose or administration of a fluid bolus of 30 ml/kg. Infections due to multiresistant microorganisms were significantly more frequent in public hospitals than in private ones.

**Table 2:** Patient characteristics at admission, outcomes, and hospitals’ characteristics

	Public hospitals’ ICUs = 23	Private hospitals’ ICUs = 26	Missing values	*p* value
Number of patients	367 (45)	442 (55)		
Patient characteristics				
Patient origin upon entry to study			23	0.20
Community	192/354 (54)	239/432 (55)		
Hospital	113/354 (32)	138/432 (32)		
ICU	40/354 (11)	40/432 (9)		
Third-level institution	5/354 (1)	14/432 (3)		
Unknown	4/354 (1)	1/432 (0)		
APACHE II	20 ± 9 *N* = 323	18 ± 7 *N* = 419	67	< 0.01
SOFA	8 [5–11] *N* = 354	6 [4–9] *N* = 433	22	< 0.01
Lactate (mmol/L)	2 [2–4] *N* = 315	2 [1–3] *N* = 404	90	< 0.01
Admission with septic shock	172/367 (47)	155/442 (35)	0	< 0.01
Use of mechanical ventilation	257/367 (70)	211/440 (48)	2	< 0.01
Requirement of a fluid bolus of 30 ml/kg	264/367 (72)	309/442 (70)	0	0.47
Time to first antibiotic dose (hours)	2 [1–3] *N* = 301	2 [1–3] *N* = 398	10	0.47
Antibiotic administration			110	0.31
Inadequate	39 (13)	43 (11)		
Adequate	168 (56)	245 (62)		
Negative cultures (%)	94 (31)	110 (28)		
Infections by highly resistant microorganisms†	93/367 (26)	85/442 (19)	0	0.02
Patient outcomes				
Length of mechanical ventilation (days)	8 [4–15] *N* = 338	8 [3–16] *N* = 407	64	0.44
Length of ICU stay (days)	10 [4–20] *N* = 359	9 [4–18] *N* = 430	20	0.20
Length of hospital stay (days)	18 [9–37] *N* = 337	19 [9–31] *N* = 390	82	0.22
28-day mortality	153/367 (42)	107/442 (24)	0	< 0.01
Hospital mortality	172/367 (47)	131/442 (30)	0	< 0.01

Data are presented as *n* (%), mean ± standard deviation, or mdn [0.25–0.75] percentiles, unless specified

*APACHE II* Acute Physiologic And Chronic Health Evaluation score, *SOFA* Sequential Organ Failure Assessment

†Highly resistant microorganisms include methicillin-resistant *Staphylococcus aureus*, vancomycin-resistant *Enterococcus*, *Pseudomonas aeruginosa, Acinetobacter baumannii*, and β-lactamase-producing *Klebsiellae*

**Fig. 2:**
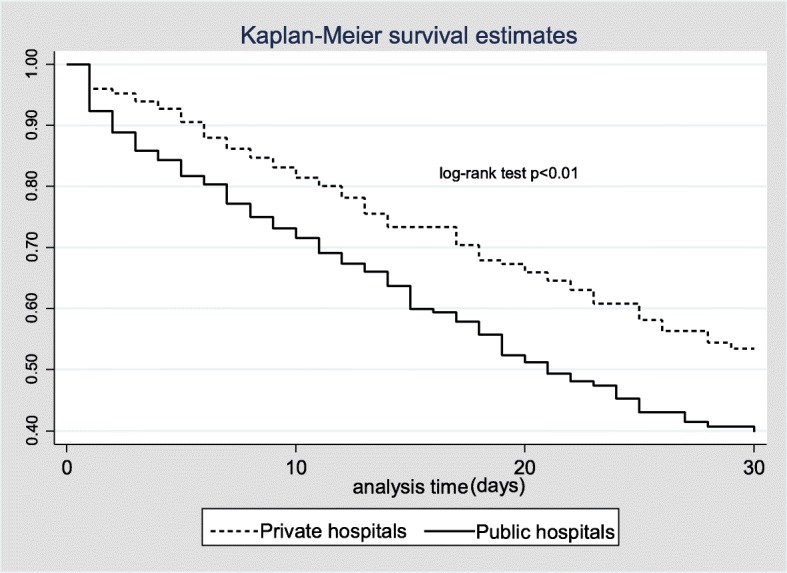
Differences in time to survival in patients admitted to public and private hospitals

Twenty-eight-day mortality was 42% in public hospitals (vs. 24% in private; *p* < 0.01); hospital mortality was respectively 47% vs. 30% (*p* < 0.01); Kaplan-Meier estimates of survival were also different (log-rank test *p* < 0.01; Fig. 2). No differences in the length of mechanical ventilation or of ICU and hospital stay were recorded.

ICU bed number was similar in public and private hospitals, but human resources (nurse-to-patient-ratio, number of board-certified specialists and of residents) were higher in public hospitals (Additional file 1: Table S1).

Localization of sites of infection was similar across groups (Additional file 1: Table S2).

With regard to independent determinants of mortality (Table 3 and Additional file 1: Table S3), variables related to higher comorbid disease, and higher severity of acute disease such as SOFA score, increasing lactate levels, requirement of mechanical ventilation, and admission to a public hospital, were associated to higher mortality, with excellent model calibration and discrimination. This is shown in model 1. However, when other relevant covariates such as duration of previous symptoms and infection by multiresistant microorganisms were tested in the model, admission to a public hospital ceased to have a significant association with mortality—acting as a correlate for the last two variables (Table 3). These results were consistent after performing multiple imputation for missing values in the variables of interest (Additional file 1: Tables S4 and S5).

**Table 3:** Independent determinants of mortality by means of logistic regression analysis

Variable	Multivariable analysis OR [95%CI]	
	Model 1	Model 2
Charlson score	1.22 [1.13–1.33] *p* < 0.01	1.29 [1.16–1.42] *p* < 0.01
Previous health state (EQ-VAS)		
Previous duration of disease		1.005 [1.004–1.010] *p* = 0.047
Lactate (mmol/L)	1.20 [1.10–1.31] *p* < 0.01	1.28 [1.15–1.41] *p* < 0.01
SOFA 24 h	1.13 [1.07–1.20] *p* < 0.01	1.14 [1.06–1.22] *p* < 0.01
Mechanical ventilation utilization	8.61 [5.21–14.23] *p* < 0.01	12.91 [6.84–24.35] *p* < 0.01
Highly resistant microorganisms†		1.76 [1.05–2.95] *p* = 0.032
Admission to a public hospital	1.47 [1.00–2.17] *p* = 0.048	1.24 [.78–1.96] *p* = 0.360

Model calibration and discrimination: model 1, which includes public hospital as a variable, has an area under the receiver operating characteristic (ROC) curve of 0.83 [0.80–0.86], with a Hosmer-Lemeshow test of 0.99. For model 2, values are 0.86 [0.83–0.89], respectively. Bivariate analysis is presented in Additional file 1: Table S2

*OR* odds ratio, *CI* confidence interval, *EQ-VAS* EuroQol visual analogue scale (from 100 points [best health state to 0 worst] self-evaluated health state, previously to the diagnosis of sepsis), *SOFA* Sequential Organ Failure Assessment

†Highly resistant microorganisms include methicillin-resistant *S. aureus*, vancomycin-resistant *Enterococcus*, *P. aeruginosa, A. baumannii*, and β-lactamase-producing *Klebsiellae*

## Discussion

In this large, prospective cohort study carried out in Argentina in patients with sepsis and septic shock, admission to a public hospital was associated with higher mortality compared to admission to a private institution. In LMICs, this phenomenon has already been reported in sepsis and critically ill obstetric patients [23–25]. The difference in the main outcome (47% vs. 30% mortality for public vs. private hospitals) was striking and reflected that patients admitted to public hospitals—although younger—were more acutely ill, had more underlying diseases, had a longer period of sepsis symptoms, and suffered from more frequent infections by highly resistant microorganisms.

Socioeconomic differences might have impacted outcomes indirectly since admission to hospitals belonging to the public health subsector can be considered a proxy of low SES, given that they serve uninsured, socially disadvantaged groups. In accordance with this, patients from public hospitals in our study had significantly fewer years of education (a difference of 5 years), lower BMI, more alcohol-related problems, and higher rates of unemployment or illegal jobs. Furthermore, public hospitals are usually underfinanced by national or local health ministries [29]. Differential outcomes related to SES are not exclusive to LMICs: in HICs like England, a higher “deprivation index” (calculated after education, employment, income, disabilities, and other variables) has been related to increased ICU mortality [30]. In the USA, in a nationwide retrospective cohort analysis of patients with sepsis, those with the lowest median income level were younger, less likely to be Caucasian, had lower health insurance coverage, and had higher mortality, compared to residents of the highest income quartile [31]. In other HICs, low SES has also been associated with more severe disease upon ICU admission and higher comorbid conditions [32, 33].

The fact that patients from public hospitals had experienced 48 h of sepsis-related symptoms before having been admitted (vs. 24 h from those in private institutions) is remarkable and deserves close attention. This finding may be explained using the conceptual framework of “the three-delay model” developed to explain the obstacles in obtaining appropriate obstetric care in low-income countries [34]. This model has recently been applied to evaluate emergency care given that LMICs bear a high burden of emergencies [35]. The first barrier that produces a delay in diagnosis and treatment is patient lack of awareness of a potentially severe condition; it is ascribed to educational and cultural factors [34, 35]. In our study, educational achievements were notably lower in patients in the public health subsector, likely contributing to their inability to recognize illness severity and subsequently to the higher frequency of admission with septic shock.

Patients with low education might also be unaware that they have chronic medical conditions. In this study, despite their significantly higher Charlson score, patients from public hospitals rated self-perception of comorbidities equal to that of patients from private institutions. A “dose-response” relationship between educational attainment and health is well-established in the literature [16, 36].

In the 3-delay conceptual framework, the second delay is access to healthcare facilities, which includes geographical distance and transportation issues. Of note, in Argentina, patients can be admitted directly to public hospitals without previous evaluation in clinics. We did not find differences in home-to-hospital distance between groups, perhaps because most patients lived in big cities, reflecting the unbalanced distribution of population in Argentina—which is 92% urban [37]. However, availability of patient transportation might have differed between both types of hospitals; it has been considered a surrogate of health insurance [38].

Lastly, it is worth mentioning that the arrival at a healthcare venue does not imply receiving immediate diagnosis and treatment. Patients in public hospitals reported antecedent visits to health clinics without inpatient admission more frequently than patients in private hospitals. Lack of recognition of community-acquired sepsis might have contributed to this.

Lack of awareness of sepsis in healthcare providers has been reported in an LMIC like Brazil, where patients admitted to public hospitals with sepsis exhibited higher mortality compared to those admitted to private hospitals. The authors ascribed this worsened prognosis to delay in diagnosis and thereafter in receiving appropriate treatment [39]. General lack of knowledge of physicians and healthcare personnel about sepsis occurs worldwide [40–42]; these situations might lead to delayed treatment and progression to more severe forms of disease (i.e., septic shock). Notwithstanding this, regarding sepsis management in the ICU, patients obtained similar treatment in both types of hospitals. Indeed, median time to antibiotic administration at sepsis recognition was 2 h, in keeping with the 3-h bundle of the Surviving Sepsis Campaign; still, this was longer than the 1-h period recently recommended, which underscores the concept of sepsis as a medical emergency [43–45]. Initial fluid management (administration of a 30 ml/kg bolus) and the proportion of patients with negative cultures/adequate/inadequate antibiotics also did not differ. Infections by multiresistant microorganisms were more frequent in public hospitals, and this was an independent predictor of mortality. Most unexpectedly, this complication could not be attributed to personnel shortage as the public hospitals recorded higher physician-to-patient and nurse-to-patient ratios. However, turnover is frequent in the public system, as described in other LMICs [25], and may be associated with inappropriate care. Beyond the numbers of healthcare personnel, processes of care are what seem to impact clinical outcomes [46]. We did not record the use of protocols in this study; nevertheless, in Latin-American ICUs, their utilization is suboptimal [17]. A differential adoption of hand-washing practices or other quality improvement initiatives between both types of institutions might explain our findings. What is more, in a recent survey of 735 Latin-American intensivists, the evaluation of satisfactory conditions to treat septic shock was lower in public hospitals due to insufficient technology, laboratory support, imaging resources, and drug availability [47].

Solutions are complex, since educational level and occupation/employment are components of SES together with income and wealth [48], all factors deeply affected in the population served by public institutions. Nevertheless, some factors are modifiable, thus establishing new targets for public health intervention. The World Health Organization has instructed its country members to increase awareness of sepsis both in the general public and in healthcare workers through public campaigns, educational activities, safety patient projects, and advocacy efforts―highlighting sepsis as a preventable but time-sensitive disease [49, 50]. Such initiatives have already been implemented in many countries [51, 52], yet not in Argentina. To date in Argentina, there have been campaigns targeting vaccinations, AIDS, and seasonal infectious diseases like measles, influenza, hantavirus pulmonary syndrome, dengue, zika, and leptospirosis via TV advertisements, billboards, street advertising, and outreach programs [53]. As the WHO recommends, health authorities could increase awareness by introducing the term “sepsis” as a comprehensive concept and using it to communicate with population at large as well as patients, families, and healthcare workers. The WHO also suggests fostering activities on September 13, which has been established as World Sepsis Day [49].

Within the ICU, the development of local quality improvement programs to increase compliance with the Surviving Sepsis Campaign bundles might improve the situation since they are associated with decreased mortality when implemented [43, 54, 55]. A word of caution is necessary since belonging to a South American country, a public hospital, or an LMIC is related to low compliance with campaign bundles [23, 56]. This low compliance underscores deep deficiencies in terms of resources, training, ICU beds, drugs and devices, and low adherence to protocols [17]. It is our hope that this study will be considered a step towards addressing the problem of sepsis in Argentina.

This study has limitations. Center participation was voluntary and might bias results. However, we sampled 7.2% of all Argentine ICUs (49/681), and the proportion between public and private institutions was consistent with the national distribution. We did not measure income directly, but we recorded other determinants of SES such as education level and occupation. We were not able to demonstrate an independent effect of inequity markers on mortality, such as education or employment status. However, it is possible that these variables reflecting deprivation states need to be assessed comprehensively in an index including further indicators, instead of isolated variables [30, 32, 33]. We consider that solely being admitted to a public hospital reflects the variables related to health inequities. Our data might not be generalizable to septic patients admitted in other settings such as EDs or wards.

Strengths of the study consist in its prospective design and the comprehensive evaluation of relevant variables related to sepsis epidemiology, treatment, and outcome. Additionally, this is the first study to specifically target data about SES in ICU patients with sepsis.

## Conclusion

In this prospective cohort study from Argentina, patients with sepsis and septic shock admitted to public hospitals had remarkably higher mortality in comparison to those in private institutions. Patients from public hospitals—while younger—were more acutely ill on admission and showed more comorbid conditions; these factors are consistently associated with worse outcomes in sepsis. In addition, a lack of recognition of severity of disease—both by patients and healthcare providers in the public system—might have extended symptom duration and subsequently delayed diagnosis and timely hospital admission. Notwithstanding, sepsis management and human resources were similar in both types of hospitals; however, infections by multiresistant microorganisms, usually associated with increased mortality, were more frequent in public hospitals.

These issues highlight the need for education to raise awareness of sepsis, both within the community and for healthcare personnel. It is also imperative to improve processes of care in the ICU, which are notoriously deficient in Latin-America.

## Additional file

Additional file 1: **Table S1.** Characteristics of the participating hospitals. **Table S2.** Localization of the sites of infection in patients in public and private hospitals. **Table S3.** Bivariable analysis for mortality. **Table S4.** Full multivariable logistic regression model 1 after multiple imputation. **Table S5.** Full multivariable logistic regression model 2 after multiple imputation. (DOCX 24 kb)

## Data Availability

The datasets used/analyzed during the current study are available from the corresponding author on reasonable request.

## Abbreviations

APACHE II: Acute Physiology And Chronic Health Evaluation II
ARDS: Acute respiratory distress syndrome
BMI: Body mass index
EQ-VAS: Euro-QoL visual analogue scale
GNI: Gross National Income
ICU: Intensive care unit
LMICs: Low- and middle-income countries
SATI: Sociedad Argentina de Terapia Intensiva (Argentine Society of Intensive Care)
SES: Socioeconomic status
SOFA: Sequential Organ Failure Assessment
