# Supplementary material for: Health inequities in the diagnosis and outcome of sepsis in Argentina: a prospective cohort study
Source: Crit Care. 2019 Jul 9;23:250. doi: 10.1186/s13054-019-2522-6 (PMC6615149; doi:10.1186/s13054-019-2522-6)
Supplement: Supplementary file 1 — Table S1. Characteristics of the participating hospitals. Table S2. Localization of the sites of infection in patients in public and private hospitals. Table S3. Bivariable analysis for mortality. Table S4. Full multivariable logistic regression model 1 after multiple imputation. Table S5. Full multivariable logistic regression model 2 after multiple imputation. (DOCX 24 kb) [file 13054_2019_2522_MOESM1_ESM.docx]

**Health inequities in the diagnosis and outcome of sepsis in Argentina: A prospective cohort study**

**Online Supplemental Material**

Table S1. Characteristics of the participating hospitals

| Variable | Public  N=23 | Private  N=26 | Missing  values | P value |
| --- | --- | --- | --- | --- |
| Number of ICU beds | 13±5 | 15±6 | 5 | 0.13 |
| Nurse:patient ratio (%)  1:1-2  1:3-4  1:4-5 | 15 (66)  8 (34)  0 (0) | 17 (65)  8 (30)  1 (5) | 5 | <0.01 |
| Board-certified critical care specialists | 11 [8-30] | 8 [6-16] | 3 | <0.01 |
| Residency in critical care medicine | 5 [2-12] | 4 [0-10] | 0 | <0.01 |

Data are presented as n (%), mean ± standard deviation, or mdn [0.25-0.75] percentiles], unless specified.

Table S2. Localization of the sites of infection in patients in public and private hospitals

| Site of infection | Public hospitals | Private hospitals |
| --- | --- | --- |
| Number of ICUs | 23 | 26 |
| Number of patients | 367 (45) | 442 (55) |
| Localization of infection* |  |  |
| Respiratory | 190/357 (53) | 198/440 (45) |
| Intraabdominal | 70/357 (21) | 107/440 (25) |
| Urinary tract | 19/357 (5e) | 52/440 (12) |
| Osteomyelitis/arthritis/soft tissue/fasciitis | 18/357 (5) | 28/440 (7) |
| Catheter-related bacteremia | 13/357 (4) | 11/440 (3) |
| Primary bacteremia | 10/357 (3) | 4/440 (1) |
| Meningitis (Community and post-neurosurgery) | 6/357 (2) | 12/440 (2) |
| Endocarditis/intravascular implanted devices | 6/357 (1) | 9/440 (2) |
| Mediastinitis | 3/357 (1) | 5/440 (1) |
| Post-operative wound site | 5/357 (1) | 5/440 (1) |
| Unknown source | 17/357 (5) | 13/440 (3) |

Data are presented as n (%).Percentages sum up higher than 100 per group because some patients had more than 1 site of infection. The site of infection was not recorded in 12 patients.

*P value=0.106 for the comparison of the distribution of site of infection between groups.

Table S3: Bivariable analysis for mortality

| Variable | Bivariable analysis  OR [95%CI] | P value |
| --- | --- | --- |
| Age | 1.01 [1.01-1.02] | p <0.01 |
| Female gender | 0.78 [0.58-1.04] | 0.09 |
| Charlson score | 1.21 [1.13-1.29] | p <0.01 |
| Previous healthstate  (EQ-VAS) * | 0.98 [0.97-0.99] | p <0.01 |
| Previous duration of disease | 1.005 [1.002-1.008] | p <0.01 |
| Body mass index (kg/m^2^) | 1.02 [1.00-1.04] | 0.03 |
| Self-perception of previous health state EQ-VAS* | 0.99 [0.98-0.99] | P<0.01 |
| Smoking habit | 1.21 [0.88-1.66] | 0.24 |
| Alcohol-related problem | 1.37 [0.91-2.07] | 0.14 |
| Distance to the hospital (km) | 1.00 [0.99-1.00] | 0.58 |
|  |  |  |
| Previous evaluation in other healthcare venue** | 1.02 [0.73-1.34] | 0.87 |
| Admission to a public hospital | 2.09 [1.57-2.80] | p <0.01 |
| APACHE II | 1.10 [1.07-1.12] | p <0.01 |
| Lactate (mmol/L) | 1.22 [1.14-1.31] | p <0.01 |
| SOFA24hs | 1.26 [1.20-1.32] | p <0.01 |
| Mechanical ventilation utilization | 9.16 [6.02-14.00] | p <0.01 |
| Highly-resistant microorganisms† | 1.55 [1.11-2.17] | p <0.01 |

*EQ-VAS: EuroQol Visual analogue scale (From 100 points [best health state to 0 worst] self-evaluated health state, previously to the diagnosis of sepsis)

**Includes primary practices, lower-complexity hospitals, or major hospitals

Table S4. Full multivariable logistic regression Model 1 after multiple imputation

|  | OR [95%CI] | P value |
| --- | --- | --- |
| Lactate (mmol/L) | 1.16 [1.06-1.28] | p<0.01 |
| Charlson score | 1.22 [1.13-1.32] | p<0.01 |
| SOFA24hs | 1.14 [1.08-1.20] | p<0.01 |
| Mechanical ventilation utilization | 7.68 [4.73-12.45] | p<0.01 |
| Admission to a public hospital | 1.44 [1.00-2.07] | 0.047 |
|  |  |  |

To avoid bias introduced by missing data, and assuming that data were missing at random, the analysis of the predictors of the primary outcome was replicated after multiple imputation. This analysis was conducted using the method of predictive mean matching for continuous variables and logistic regression for categorical variables

To fit the first logistic regression model for the dependent variable hospital mortality (Model 1, Table 3 of the main body of the manuscript) we considered lactate as the main imputed variable. The variables also considered were mechanical ventilation utilization and admission to a public hospital.

The number of imputations chosen for the variable lactate was 20, 40, 60 and 80. The multivariable model was reproduced in the 4 datasets after multiple imputation and the results were pooled. The results were consistent with the original Model 1 shown in Table 3 in the main body of the manuscript.

Table S5. Full multivariable logistic regression Model 2 after multiple imputation

|  | OR [95%CI] | P value |
| --- | --- | --- |
| Lactate (mmol/L) | 1.24 [1.10-1.38] | p<0.01 |
| Charlson score | 1.26 [1.14-1.38] | p<0.01 |
| SOFA24hs | 1.15 [1.08-1.23] | p<0.01 |
| Mechanical ventilation utilization | 11.82 [6.43-21.69] | p<0.01 |
| Previous duration of disease | 1.01 [1.00-1.01] | 0.022 |
| Highly-resistant microorganisms† | 1.67 [1.01-2.76] | 0.044 |

† Highly-resistant microorganisms include methicillin-resistant S.aureus, vancomycin-resistant Enterococcus, P. aeruginosa, A. baumannii, and β-lactamase-producing Klebsiellae.

To avoid bias introduced by missing data, and assuming that data were missing at random, the analysis of the predictors of the primary outcome was replicated after multiple imputation. This analysis was conducted using the method of predictive mean matching for continuous variables and logistic regression for categorical variables

To fit the second regression model for the dependent variable hospital mortality (Model 2, Table 3 of the main body of the manuscript) we considered lactate as the main imputed variable (90 missing values). The variables also considered in were: Charlson score, SOFA24hs, mechanical ventilation utilization and duration of previous disease symptoms (hours), and presence of infection with highly-resistant microorganisms.

The number of imputations chosen for the variable lactate was 20, 40, 60 and 80. The multivariable model was reproduced in the 4 datasets after multiple imputation and the results were pooled. The results were consistent with the original Model 2 shown in Table 3 in the main body of the manuscript.
